# Risk factors for virologic failure and persistent low-level viremia in people with HIV experiencing low-level viremia: Chongqing ART cohort study, 2019–2023

**DOI:** 10.3389/fmed.2025.1660030

**Published:** 2025-09-18

**Authors:** Huizheng Zhang, Jinjin Liu, Zhen Zhang, Yuqi Huo, Wanting Zeng

**Affiliations:** ^1^Department of Clinical Laboratory, Chongqing Public Health Medical Center, Chongqing, China; ^2^Affiliated Infectious Diseases Hospital of Zhengzhou University (Henan Infectious Diseases Hospital, The Sixth People’s Hospital of Zhengzhou), Zhengzhou, China; ^3^Department of Pharmacy, Chongqing Public Health Medical Center, Chongqing, China; ^4^Department of Otolaryngology Head and Neck Surgery, Daping Hospital, Army Medical University, Chongqing, China

**Keywords:** risk factors, low-level viremia, virologic failure, persistent low-level, Chongqing

## Abstract

**Background:**

Low-level viremia (LLV) during effective antiretroviral therapy (ART) presents ongoing management challenges globally, with reported prevalence rates of 10–46% in resource-limited settings. The clinical significance of LLV remains controversial: while some studies demonstrate that viral load (VL) levels exceeding 200 copies/mL predict virologic failure (VF), others report no significant association. This uncertainty underscores the need for clearer risk stratification in diverse clinical settings.

**Objective:**

To investigate risk factors for VF and persistent low-level viremia (pLLV) in HIV-1-infected individuals experiencing LLV.

**Design:**

A retrospective cohort study between January 2019 and December 2023, consisting of 1,214 individuals with LLV (defined as plasma HIV-1 RNA levels of 50–999 copies/mL detected at two consecutive time points following previously undetected viral loads) at a large specialized hospital in Chongqing, China.

**Methods:**

Clinical data, including demographics, ART regimens, adherence, baseline viral load (VL), CD4 + T-cell counts, and LLV characteristics, were extracted from medical records. Univariate and multivariate logistic regression models were used to identify factors associated with VF (defined as one or more HIV VLs of ≥1,000 copies/mL) and pLLV (defined as at least three consecutive measurements of VL within the range of 50 to 999 copies/mL), with adjustments for potential confounders.

**Results:**

Among 1,214 participants with LLV, 2.64% (32/1,214) developed VF, and 28.09% (341/1,214) developed pLLV. Protective factors against VF included baseline VL < 1,000 copies/mL (adjusted odds ratio [aOR] = 0.100, 95%CI: 0.013–0.765) and VL < 200 copies/mL during LLV (aOR = 0.157, 95%CI: 0.071–0.540). Viral blips (transient LLV) independently predicted VF (aOR = 4.6775, 95%CI: 1.392–15.704). For pLLV, baseline VL < 1,000 copies/mL remained protective (aOR = 0.569, 95% CI: 0.329–0.984), while primary education or lower was a risk factor (aOR = 2.052, 95%CI: 1.014–4.194).

**Conclusion:**

VL levels during LLV and baseline VL predict VF risk, emphasizing the need for vigilant VL monitoring and adherence support.

## Introduction

As of December 2023, an estimated 3.99 million individuals globally were living with HIV (1, 2), with China accounting for 1.29 million reported cases. Chongqing, the site of China’s first documented AIDS case in 1993, had registered approximately 68,000 people with HIV by October 2023. Of these, approximately 62,000 (91.2%) received antiretroviral therapy (ART). During the first 10 months of 2023, the region reported 7,154 new diagnoses and 2,729 deaths among people with AIDS.

ART has transformed HIV management through plasma viral load (VL) suppression, immune function preservation, and delayed disease progression, significantly improving survival of people receiving treatment while reducing transmission risks. Nevertheless, ART regimens fail to achieve complete viral replication suppression in some patients, leading to two distinct virological patterns: (1) persistent low-level viremia (pLLV), defined as sustained VL ranging from 20 to 999 copies/mL, depending on the diagnostic thresholds, and (2) transient viremic episodes, termed blips. Current guidelines show significant variation in LLV definitions: the European AIDS Clinical Society (EACS) uses 20–50 copies/mL (3–5), while U. S. DHHS recommends 50–200 copies/mL (6–9) (prevalence 3.5–9.9%) (10) and WHO adopts 50–999 copies/mL for resource-limited settings (11–14) (prevalence 10–46%) (15, 16). No existing guidelines recommend ART modification specifically for LLV management.

The pathophysiology of LLV remains incompletely characterized, with two predominant mechanistic theories: (1) activation of latent viral reservoirs, and (2) ongoing viral production from pharmacological sanctuary sites. Clinical evidence regarding LLV’s association with VF shows persistent contradictions. While VL levels exceeding 200 copies/mL demonstrate predictive value for VF in multiple studies (15, 17, 18), other investigations report no significant association (1). Similarly, the clinical significance of blips and pLLV remains controversial, though sustained LLV (50–200 copies/mL) may elevate VF risk according to some cohort studies (4, 19–22).

This retrospective analysis of longitudinal data from a large specialized hospital in Chongqing, China, addresses two critical gaps: (1) whether specific LLV types (pLLV vs. blips) predict subsequent VF, and (2) which clinical factors predispose LLV patients to VF. Our results provide actionable insights for optimizing ART strategies to achieve early virological suppression, particularly in resource-limited clinical environments.

## Methods

### Study population

This retrospective study analyzed a cohort of individuals receiving treatment for HIV-1 with long-term follow-up from 2019 to 2023 at a large specialized hospital in Chongqing, China. All individuals received ART following the Chinese HIV/AIDS diagnosis and treatment guidelines. Follow-up visits occurred every 3 to 6 months to assess VL, CD4 + T-cell counts, and other routine clinical parameters. Sociodemographic data, including gender, age at HIV diagnosis, marital status, education level, ethnicity, and mode of transmission, were collected. Clinical information, such as ART duration (months), time from diagnosis to ART initiation, ART regimen, and laboratory findings, including VL, CD4 + T-cell count, was retrieved from clinical follow-up records. Medication adherence was assessed using follow-up case notes and pharmacy administration records. LLV was defined as the occurrence of one (blips) or two consecutive VL measurements of 50–999 copies/mL after virologic suppression while pLLV was defined as three or more consecutive VLs of 50–999 copies/mL, at least 1 month apart (1); VF was defined as one or more HIV VLs of ≥1,000 copies/mL; and virological suppression was defined as VL < 50 copies/mL. Baseline refers to clinical and laboratory parameters measured at the initiation of antiretroviral therapy (ART), including but not limited to plasma HIV RNA VL, CD4 + T-cell count, and initial ART regimen. Patients were excluded if they: (1) had an insufficient number of post-baseline viral load measurements (< 3) during follow-up, or (2) had no VL values meeting the pre-specified LLV definition (i.e., detectable viremia between 50 and 999 copies/mL after achieving virological suppression).

### Statistical analysis

Collected data were organized using Excel and analyzed with SPSS software (version 25). Quantitative variables were expressed as mean ± standard deviation (X ± S), with group comparisons performed using the independent two-sample t-test or analysis of variance (ANOVA). Qualitative variables were presented as proportions or component ratios, with statistical comparisons conducted using the χ^2^ test, Fisher’s exact probability method, or the Kruskal-Wallis test, as appropriate. Logistic regression models were employed for univariate and multivariate analyses to identify potential risk factors, with a significance level set at *α* = 0.05.

## Results

### Population characteristics and clinical features

A total of 1,214 people living with HIV with LLV were included. The median age was 42 (IQR, 31–56), and 79.74% (968/1,214) were male. Demographics included 34.93% (424/1,214) married, 28.67% (348/1,214) single, and 60.96% (740/1,214) Han Chinese. Transmission routes were predominantly heterosexual contact (44.89%, 545/1,214), followed by men who have sex with men (MSM) (9.14%, 111/1,214) and drug use (1.07%, 13/1,214). Median baseline VL was 74,850 copies/mL (IQR, 330–487,000), and median CD4 + nadir T-cell counts during ART were 146 cells/μL (IQR,56–254) and 157 cells/μL (IQR,61–297) at baseline. CD4 + T-cell counts< 200 cells/μL at baseline occurred in 59.56% (723/1,214). Median VL during LLV was 92 copies/mL (IQR, 67–146), with 83.53% (1,014/1,214) < 200 copies/mL, 10.87% (132/1,214) had a viral load of 200–400 copies/mL, and 5.60% (68/1,214) had a viral load of 401–999 copies/mL. Median LLV duration was 14 months (IQR, 10.00–21.00). Most patients (90.94%, 1,104/1,214) initiated ART within 1 year of diagnosis. NNRTI-based regimens predominated (67.38%, 818/1,214), followed by INSTI-based (19.85%, 241/1,214) and PI-based regimens (5.19%, 63/1,214). Blips occurred in 67.71% (822/1,214) and pLLV in 32.29% (392/1,214). During LLV, 21.33% (259/1,214) modified ART regimens. Medication adherence was high (96.21%, 1,168/1,214). Comprehensive data are presented in Table 1.

**Table 1:** Characteristics of people living with HIV (PLWH) who presented with low-level viremia in Chongqing, 2019–2023.

Variables	ALL		VF				pLLV			
	(*N* = 1,214)		(*N* = 32)				(*N* = 341)			
	*N*	%	*N*	%	*χ^2^/t*	*p*	*N*	%	*χ^2^/t*	*p*
Sex	1,214	100.00%	32	2.64%			341	28.09%		
Male	968	79.74%	26	2.69%	0.047	0.829	276	28.51%	0.424	0.515
Female	246	20.26%	6	2.44%			65	26.42%		
Age at diagnosis, years, median, (IQR)	42 (31, 56)		41.5 (29.25, 59.75)		0.093	0.926	46.00 (35.00, 59.00)		−4.415	0.000
<20	14	1.15%	1	7.14%	2.526	0.773	6	42.86%	21.333	0.001
20–29	240	19.77%	7	2.92%			47	19.58%		
30–39	298	24.55%	7	2.35%			71	23.83%		
40–49	211	17.38%	5	2.37%			66	31.28%		
50–59	219	18.04%	4	1.83%			68	31.05%		
≥60	230	18.95%	8	3.48%			82	35.65%		
Marital status										
Married	424	34.93%	13	3.07%	6.984	0.137	119	28.07%	9.863	0.043
Unmarried	225	18.53%	10	4.44%			55	24.44%		
Widowed	47	3.87%	2	4.26%			22	46.81%		
Divorced	76	6.26%	1	1.32%			23	30.26%		
Unknown	442	36.41%	6	1.36%			122	27.60%		
Ethnicity										
Han	740	60.96%	25	3.38%	4.203	0.122	205	27.70%	0.719	0.698
Other	15	1.24%	0	0.00%			3	20.00%		
Unknown	459	37.81%	7	1.53%			133	28.98%		
CD4 + nadir during ART (cells/μL)	1,212	99.84%	32	2.64%			340	28.05%		
CD4 + nadir during ART, median (IQR)	146.00 (56.00/254.75)		112.50 (39.50/247.25)		0.563	0.574	130.00 (51.00/238.75)		0.563	0.574
HIV transmission route										
HSX	545	44.89%	18	3.30%	3.064	0.382	165	30.28%	3.310	0.346
MSM	111	9.14%	4	3.60%			29	26.13%		
IDU	13	1.07%	0	0.00%			5	38.46%		
Unknown	545	44.89%	10	1.83%			142	26.06%		
Degree of education										
Primary school or below	163	13.43%	7	4.29%	3.340	0.342	62	38.04%	12.008	0.007
Junior school	189	15.57%	4	2.12%			57	30.16%		
College or above	96	7.91%	4	4.17%			20	20.83%		
Unknown	766	63.10%	17	2.22%			202	26.37%		
Baseline VL (copies/mL)	1,214	100.00%	32	2.64%			341	28.09%		
Baseline VL, median (IQR)	74850.00 (330.75/487000.00)		104000.00 (24900.00/510000.00)		0.784	0.433	152000.00 (1044.00/797224.00)		0.784	0.433
Log (Baseline VL)	1,214	100.00%	32	2.64%			341	28.09%		
<3	342	28.17%	1	0.29%	12.101	0.002	85	24.85%	14.351	0.001
3–5	306	25.21%	14	4.58%			68	22.22%		
≥5	566	46.62%	17	3.00%			188	33.22%		
Baseline CD4 + T cell count (cells/mL)	1,212	99.84%	32	2.64%			340	28.05%		
Baseline CD4 + T cell count, median (IQR)	157.00 (61.00/297.00)		130.00 (40.50/298.25)		0.075	0.940	149.00 (53.00/279.25)		0.075	0.940
<200	723	59.56%	19	2.63%	3.743	0.291	211	29.18%	6.248	0.100
200–349	273	22.49%	9	3.30%			72	26.37%		
350–499	151	12.44%	1	0.66%			33	21.85%		
≥500	65	5.35%	3	4.62%			24	36.92%		
HIV VL during LLV (copies/ml plasma)	1,214	100.00%	32	2.64%			341	28.09%		
Median (IQR)	92.30 (67.30/146.25)		144.50 (77.21/377.88)		−4.810	0.000	85.50 (64.30/134.23)		−4.810	0.000
<200	1,014	83.53%	18	1.78%	22.125	0.000	292	28.80%	1.540	0.463
200–400	132	10.87%	7	5.30%			32	24.24%		
401–999	68	5.60%	7	10.29%			17	25.00%		
LLV duration	1,214	100.00%	32	2.64%			341	28.09%		
Time from Diagnosis to ART Initiation, Years	1,198	98.68%	31	2.59%			334	27.88%		
<1	1,104	90.94%	29	2.63%	0.507	0.776	308	27.90%	2.696	0.260
1–5	68	5.60%	1	1.47%			22	32.35%		
≥6	26	2.14%	1	3.85%			4	15.38%		
Treatment regimens	1,214	100.00%	32	2.64%			341	28.09%		
NRTI + NNRTI	818	67.38%	20	2.44%	3.342	0.502	210	25.67%	16.255	0.003
NRTI + PI	63	5.19%	2	3.17%			23	36.51%		
NRTI + INSTI	241	19.85%	5	2.07%			78	32.37%		
Other	76	6.26%	4	5.26%			20	26.32%		
Unknown	16	1.32%	1	6.25%			10	62.50%		
blips/pLLV	1,214	100.00%	32	2.64%			341	28.09%		
blips	822	67.71%	29	3.53%	7.893	0.005	28	3.41%	767.814	0.000
pLLV	392	32.29%	3	0.77%			313	79.85%		
Switching regimens	1,214	100.00%	32	2.64%			341	28.09%		
Yes	259	21.33%	4	1.54%	1.731	0.421	69	26.64%	4.700	0.095
No	949	78.17%	28	2.95%			268	28.24%		
Unknown	6	0.49%	0	0.00%			4	66.67%		
ART adherence	1,214	100.00%	32	2.64%			341	28.09%		
Good	1,168	96.21%	28	2.40%	9.307	0.025	326	27.91%	6.711	0.082
Poor	30	2.47%	3	10.00%			8	26.67%		
Unmedicated	9	0.74%	1	11.11%			2	22.22%		
Unknown	7	0.58%	0	0.00%			5	71.43%		

Data are presented as *n* (%) or median (IQR); IQR, interquartile range; significance for differences was measured using the Chi-squared test, Fisher’s Exact test, or Kruskal–Wallis test. HSX, heterosexual transmission; MSM, men who have sex with men; IDU, intravenous drug use; NRTI, nucleoside reverse transcriptase inhibitors; NNRTI, non-nucleoside reverse transcriptase inhibitors; PI, protease inhibitors; INSTI, integrase strand transfer inhibitors.

### Risk factor analysis

Among 32 VF cases, χ^2^ tests identified significant associations with baseline VL (χ^2^ = 12.101, *p* = 0.002), LLV-phase VL (χ^2^ = 22.125, *p* < 0.001), blips/pLLV (χ^2^ = 7.893, *p* = 0.005), and suboptimal adherence (χ^2^ = 9.307, *p* = 0.025) (Table 1). For 341 pLLV patients, significant correlates included age (χ^2^ = 21.333, *p* = 0.001), marital status (χ^2^ = 9.863, *p* = 0.043), education (χ^2^ = 12.008, *p* = 0.007), baseline VL (χ^2^ = 14.351, *p* = 0.001), ART regimen (χ^2^ = 16.225, *p* = 0.003).

### Key subgroup differences in risk factors for virologic outcomes

Subgroup analyses stratified by transmission route (MSM vs. non-MSM) and ART regimen (INSTI-based vs. non-INSTI-based) revealed distinct patterns. In the MSM transmission subgroup (*n* = 111, 3.6% VF rate), LLV levels < 200 copies/mL (OR = 0.074, 95%CI: 0.009–0.611; *p* = 0.016) and baseline log_₁₀_ VL < 3 (OR = 0.169, 95%CI: 0.036–0.789; *p* = 0.024) were protective, though extreme OR values for blips (vs pLLV) and adherence due to small sample size limited interpretability. The non-MSM subgroup (*n* = 1,103, 2.54% VF rate) showed strong protective effects of blips (adjusted OR = 10.434, 95%CI: 1.355–80.331; *p* = 0.024) and LLV < 200 copies/mL (OR = 0.176, 95%CI: 0.035–0.896; *p* = 0.036), with baseline log_₁₀_ VL 3–5 associated with elevated VF risk (adjusted OR = 6.671, 95%CI: 1.245–35.736; *p* = 0.027); lower education correlated with pLLV, while unmarried status was protective. The INSTI-based regimen subgroup (n = 241, 2.07% VF rate) showed a protective trend for adherence (OR = 0.087, 95%CI: 0.008–0.928; *p* = 0.043) but unreliable blip results. The non-INSTI subgroup (*n* = 973, 2.77% VF rate) confirmed protective effects of baseline log₁₀ VL < 3 (OR = 0.100, 95%CI: 0.013–0.771; *p* = 0.027), LLV < 200 copies/mL (OR = 0.193, 95%CI: 0.067–0.552; *p* = 0.002), and blips (OR = 3.382, 95%CI: 1.007–11.356; *p* = 0.049) (Tables 2, 3).

**Table 2:** Effect sizes and multivariable-adjusted analysis of factors associated with VF across subgroups.

Indicator	Overall population (*n* = 1,214)	MSM transmission subgroup (*n* = 111)	Non-MSM transmission subgroup (*n* = 1,103)	INSTI subgroup (*n* = 241)	Non-INSTI subgroup (*n* = 973)
Baseline characteristics					
Sample size (VF Cases)	1,214 (32)	111 (4)	1,103 (28)	241 (5)	973 (27)
VF rate, %	2.64	3.6	2.54	2.07	2.77
Effect size of key factors, OR, 95% CI; *p*-value					
Log (Baseline VL)					
<3	0.095 (0.013–0.715; 0.022)	0.000 (0.000–.;0.998)^*^	0.189 (0.024–1.462; 0.110)	0.000 (0.000–.; 0.998)^*^	0.100 (0.013–0.771; 0.027)
3–5	1.548 (0.753–3.186; 0.235)	2.320 (0.309–17.407; 0.413)	0.782 (0.250–2.448; 0.673)	2.167 (0.352–13.348; 0.405)	1.504 (0.675–3.350; 0.318)
≥5	ref	ref	ref	ref	ref
HIV VL during LLV (copies/ml plasma)					
<200	0.157 (0.063–0.391; 0.000)	0.074 (0.009–0.611; 0.016)	0.176 (0.035–0.896; 0.036)	0.144 (0.014–1.513; 0.106)^*^	0.193 (0.067–0.552; 0.002)
200–400	0.488 (0.164–1.454; 0.198)	0.000 (0.000–.; 0.999) ^*^	0.969 (0.227–4.133; 0.966)	0.556 (0.031–9.873; 0.689)^*^	0.583 (0.170–2.001; 0.391)
401–999	ref	ref	ref	ref	ref
blips vs. pLLV	4.742 (1.436–15.663; 0.011)	79776537.950 (0.000–.; 0.998)^*^	9.764 (1.290–73.926; 0.027)	57286341.237 (0.000–.; 0.997)^*^	3.382 (1.007–11.356; 0.049)
Good adherence vs. poor adherence	0.221 (0.063–0.772; 0.018)	62736889.628 (0.000–.; 0.999)^*^	0.248 (0.052–1.169; 0.078)	0.087 (0.008–0.928; 0.043)	0.268 (0.059–1.211; 0.087)
Regimen switch vs. no switch	0.516 (0.179–1.484; 0.220)	0.988 (0.099–9.899; 0.992)	0.435 (0.099–1.920; 0.272)	0.658 (0.072–5.994; 0.710)^*^	0.507 (0.151–1.708; 0.273)
Multivariate-adjusted key variables (Adjusted OR 95% CI; *p*-value)					
Log (Baseline VL)					
<3	0.100 (0.013–0.764; 0.026)	0.000 (0.000–.; 0.995)^*^	-	0.000 (0.000–.; 0.998)^*^	0.113 (0.014–0.878; 0.037)
3–5	1.688 (0.793–3.593; 0.174)	3.194 (0.273–37.334; 0.355)	6.671 (1.245–35.736; 0.027)	1.722 (0.244–12.172; 0.586)^*^	1.733 (0.748–4.018; 0.200)
≥5	ref	ref	ref	ref	ref
HIV VL during LLV (copies/ml plasma)					
<200	0.197 (0.072–0.541; 0.002)	0.075 (0.009–0.618; 0.016)	0.330 (0.067–1.619; 0.172)	0.224 (0.015–3.360; 0.279)^*^	0.244 (0.074–0.807; 0.021)
200–400	0.594 (0.183–1.931; 0.387)	0.000 (0.000–.; 0.999)^*^	-	0.930 (0.032–27.429; 0.967)^*^	0.712 (0.184–2.757; 0.623)
401–999	ref	ref	ref	ref	ref
blips vs. pLLV	4.681 (1.394–15.722; 0.013)	52055872.888 (0.000–.; 0.998)^*^	10.434 (1.355–80.331; 0.024)	65987555.248 (0.000–.; 0.996)^*^	3.240 (0.947–11.085; 0.061)
Good adherence vs. poor adherence	0.277 (0.070–1.092; 0.067)	8718249.669 (0.000–.; 0.999)^*^	0.063 (0.005–0.786; 0.032)	0.058 (0.004–0.779; 0.032)^*^	0.360 (0.069–1.882; 0.226)
Regimen switch vs. no switch	-	-	-	0.510 (0.037–6.989; 0.614)^*^	0.516 (0.150–1.777; 0.295)

**Table 3:** Effect sizes and multivariable-adjusted analysis of factors associated with pLLV across subgroups.

Indicator	Overall population (*n* = 1,214)	MSM transmission subgroup (*n* = 111)	Non-MSM transmission subgroup (*n* = 1,103)	INSTI subgroup (*n* = 241)	Non-INSTI subgroup (*n* = 973)
Baseline characteristics					
Sample size (pLLV cases)	1,214 (341)	111 (29)	1,103 (312)	241 (78)	973 (263)
pLLV rate (%)	28.09	26.13	28.29	32.37	27.03
Effect size of key factors (OR, 95% CI; *p*-value)					
Age (years)					
<20	1.354 (0.454–4.036; 0.587)	0.333 (0.009–11.939; 0.547)	1.815 (0.111–29.596; 0.676)	0.000 (0.000–.; 1.000)^*^	-
20–29	0.440 (0.289–0.667; 0.000)	0.366 (0.021–6.226; 0.487)	0.253 (0.101–0.633; 0.003)	0.267 (0.098–0.722; 0.009)	0.507 (0.319–0.807; 0.004)
30–39	0.565 (0.386–0.825; 0.003)	0.423 (0.024–7.388; 0.556)	0.530 (0.306–0.918; 0.023)	0.358 (0.159–0.807; 0.013)	0.578 (0.371–0.899; 0.015)
40–49	0.822 (0.552–1.222; 0.332)	0.125 (0.004–3.996; 0.239)	0.958 (0.573–1.602; 0.870)	0.429 (0.171–1.073; 0.070)	0.925 (0.592–1.447; 0.734)
50–59	0.813 (0.548–1.205; 0.302)	0.000 (0.000–.; 0.999)^*^	0.961 (0.588–1.570; 0.873)	0.613 (0.264–1.424; 0.255)	0.822 (0.522–1.296; 0.400)
≥60	ref	ref	ref	ref	ref
Marital status					
Married	1.023 (0.760–1.378; 0.879)	0.889 (0.061–12.885; 0.931)	0.726 (0.381–1.387; 0.333)	0.919 (0.428–1.974; 0.829)	1.008 (0.722–1.407; 0.960)
Unmarried	0.849 (0.587–1.227; 0.383)	0.676 (0.059–7.812; 0.754)	0.369 (0.162–0.839; 0.017)	0.703 (0.314–1.575; 0.392)	0.815 (0.518–1.285; 0.380)
Widowed	2.308 (1.255–4.247; 0.007)	0.667 (0.025–18.059; 0.810)	1.400 (0.578–3.388; 0.456)	3.222 (0.676–15.352; 0.142) *	2.150 (1.080–4.280; 0.028)
Divorced	1.138 (0.669–1.938; 0.633)	-	0.749 (0.336–1.669; 0.479)	1.160 (0.412–3.266; 0.779)	0.910 (0.440–1.880; 0.795)
Unknown	ref	ref	ref	ref	ref
Degree of education					
Primary school or below	1.714 (1.202–2.444; 0.003)	2.091 (0.119–36.635; 0.614)	1.924 (1.226–3.020; 0.004)	1.271 (0.571–2.831; 0.557)	1.870 (1.240–2.820; 0.002)
Junior school	1.206 (0.850–1.710; 0.295)	0.545 (0.173–1.723; 0.302)	1.663 (1.052–2.630; 0.029)	1.434 (0.727–2.829; 0.298)	1.000 (0.640–1.560; 0.995)
College or above	0.735 (0.438–1.234; 0.244)	0.657 (0.245–1.764; 0.405)	0.908 (0.407–2.024; 0.813)	0.581 (0.258–1.309; 0.190)	0.690 (0.320–1.480; 0.340)
Unknown	ref	ref	ref	ref	ref
Log (Baseline VL)					
<3	0.665 (0.492–0.899; 0.008)	0.169 (0.036–0.789; 0.024)	0.677 (0.425–1.078; 0.101)	0.306 (0.112–0.836; 0.021)	0.720 (0.510–1.020; 0.065)
3–5	0.574 (0.417–0.792; 0.001)	0.531 (0.185–1.518; 0.237)	0.656 (0.410–1.049; 0.078)	0.580 (0.285–1.178; 0.132)	0.550 (0.370–0.810; 0.003)
≥5	ref	ref	ref	ref	ref
HIV VL during LLV (copies/ml plasma)					
<200	0.009 (0.006–0.014; 0.000)	1.370 (0.267–7.013; 0.706)	1.464 (0.647–3.314; 0.360)	1.268 (0.326–4.931; 0.732)	1.230 (0.630–2.380; 0.530)
200–400	0.923 (0.677–1.258; 0.611)	0.000 (0.000–.; 0.999)^*^	1.393 (0.542–3.576; 0.491)	1.556 (0.307–7.873; 0.593)	0.820 (0.370–1.810; 0.615)
401–999	ref	ref	ref	ref	ref
blips vs. pLLV	0.009 (0.006–0.014; 0.000)	0.010 (0.002–0.043; 0.000)	0.011 (0.006–0.021; 0.000)	0.011 (0.004–0.029; 0.000)	0.008 (0.005–0.014; 0.000)
Good adherence vs. poor adherence	1.065 (0.469–2.416; 0.881)	0.709 (0.062–8.123; 0.782)	0.735 (0.284–1.901; 0.525)	0.465 (0.092–2.361; 0.356)	1.310 (0.470–3.620; 0.595)
Regimen switch vs. No switch	0.923 (0.677–1.258; 0.611)	0.924 (0.345–2.474; 0.875)	0.947 (0.611–1.470; 0.809)	1.400 (0.760–2.570; 0.265)	0.780 (0.530–1.140; 0.195)
Multivariable-adjusted key variables (aOR, 95% CI; *p*-value)					
Age (years)					
<20	2.341 (0.305–17.955; 0.413)	0.103 (0.000–2695.309; 0.661)^*^	-	-	-
20–29	0.909 (0.414–1.997; 0.813)	0.171 (0.000–179.453; 0.618)^*^	-	-	1.322 (0.535–3.266; 0.546)
30–39	1.024 (0.507–2.070; 0.947)	0.041 (0.000–66.464; 0.397)^*^	-	-	1.177 (0.524–2.646; 0.693)
40–49	1.942 (0.939–4.015; 0.073)	0.000 (0.000–.; 0.998)^*^	-	-	2.592 (1.115–6.025; 0.027)
50–59	0.928 (0.477–1.804; 0.826)	0.000 (0.000–.; 0.999)^*^	-	-	1.126 (0.511–2.481; 0.768)
≥60	ref	ref	ref	ref	ref
Marital status					
Married	0.645 (0.340–1.224; 0.180)	6.544 (0.000–1239803.154; 0.762)^*^	-	-	0.600 (0.289–1.246; 0.171)
Unmarried	0.961 (0.458–2.018; 0.917)	63.927 (0.000–12190551.139; 0.503)^*^	-	-	0.729 (0.288–1.849; 0.506)
widowed	1.287 (0.415–3.990; 0.662)	9378.601 (0.012–7456719388.312; 0.187)^*^	-	-	0.887 (0.258–3.057; 0.850)
Divorced	0.516 (0.186–1.434; 0.205)	-	-	-	0.456 (0.121–1.714; 0.245)
Unknown	ref	ref	ref	ref	ref
Degree of education					
Primary school or below	1.869 (0.925–3.777; 0.081)	27063242050.692 (0.000–.; 0.998)^*^	2.041 (0.915–4.552; 0.081)	-	3.672 (1.573–8.573; 0.003)
Junior school	1.672 (0.836–3.343; 0.146)	0.263 (0.012–5.981; 0.402)	2.617 (1.090–6.283; 0.031)	-	1.533 (0.653–3.601; 0.327)
College or above	0.956 (0.369–2.476; 0.926)	0.023 (0.001–0.965; 0.048)	1.499 (0.381–5.904; 0.562)	-	2.293 (0.580–9.062; 0.237)
Unknown	ref	ref	ref	ref	ref
Log (Baseline VL)					
<3	0.569 (0.332–0.972; 0.039)	0.040 (0.001–1.246; 0.067)	0.589 (0.260–1.334; 0.205)	-	0.552 (0.295–1.031; 0.062)
3–5	0.615 (0.353–1.069; 0.085)	0.015 (0.000–0.644; 0.028)	0.651 (0.291–1.457; 0.296)	-	0.557 (0.284–1.095; 0.090)
≥5	ref	ref	ref	ref	ref
HIV VL during LLV (copies/ml plasma)					
<200	0.963 (0.360–2.578; 0.940)	0.326 (0.011–9.780; 0.518)	1.407 (0.343–5.769; 0.636)	-	0.930 (0.289–2.994; 0.903)
200–400	0.425 (0.135–1.338; 0.144)	0.000 (0.000–.; 0.999) ^*^	0.606 (0.124–2.964; 0.536)	-	0.285 (0.074–1.099; 0.068)
401–999	ref	ref	ref	ref	ref
blips vs. pLLV	0.008 (0.005–0.013; 0.000)	0.000 (0.000–0.021; 0.000)	0.009 (0.005–0.019; 0.000)	0.011 (0.004–0.030; 0.000)	0.006 (0.003–0.011; 0.000)
Good adherence vs. poor adherence	1.918 (0.509–7.234; 0.336)	160.536 (0.173–149336.102; 0.145)	0.740 (0.126–4.358; 0.739)	-	3.170 (0.636–15.804; 0.159)
Regimen switch vs. no switch	0.933 (0.554–1.569; 0.793)	6.248 (0.238–164.365; 0.272)	0.988 (0.473–2.064; 0.975)	-	1.048 (0.560–1.964; 0.883)

Data marked with * indicates that the data are subject to various special circumstances, including small sample sizes and abnormal data. These factors may interfere with the accuracy and reliability of the results, so comprehensive consideration and cautious interpretation are necessary.

### Multivariate analysis

Multivariate analysis demonstrated that a baseline log₁₀ VL < 3 (aOR: 0.100, 95%CI: 0.013–0.765) was a protective factor against VF compared with baseline log_₁₀_ VL > 5. Additionally, VL < 200 copies/mL during LLV (aOR: 0.196, 95% CI: 0.071–0.540) showed protective effects relative to VL > 401–999 copies/mL (Table 4). For pLLV patients, baseline log_₁₀_VL < 3 remained protective versus log_₁₀_VL > 5. Lower educational attainment (≤elementary school) emerged as a pLLV risk factor, though other demographic and treatment factors showed no independent associations (Table 5).

**Table 4:** Multifactorial analysis of VF among HIV-infected patients with low-level viremia in Chongqing, 2019–2023.

Characteristics	All					
	Total	VF	OR (95% CI)	*p*	Adjusted OR (95% Cl)	*p*
Log_10_ (Baseline VL)						
<3	342 (28.17)	1 (0.29)	0.095 (0.013–0.715)	0.022	0.100 (0.013–0.765)	0.027
3–5	306 (25.21)	14 (4.58)	1.548 (0.753–3.186)	0.235	1.691 (0.795–3.600)	0.173
≥5	566 (46.62)	17 (3.00)	ref		ref	
VL of LLV (copies/ml)						
<200	1,014 (83.53)	18 (1.78)	0.157 (0.063–0.391)	0.000	0.196 (0.071–0.540)	0.002
200–400	132 (10.87)	7 (5.30)	0.488 (0.164–1.454)	0.198	0.594 (0.183–1.931)	0.387
401–999	68 (5.60)	7 (10.29)	ref		ref	
blips/pLLV						
blips	822 (67.71)	29 (3.53)	4.742 (1.436–15.663)	0.011	4.675 (1.392–15.704)	0.013
pLLV	392 (32.29)	3 (0.77)	ref		ref	
Treatment regimens						
NRTI + NNRTI	818 (67.38)	20 (2.44)	0.451 (0.150–1.356)	0.156	-	-
NRTI + PI	63 (5.19)	2 (3.17)	0.590 (0.104–3.333)	0.551	-	-
NRTI + INSTI	241 (19.85)	5 (2.07)	0.381 (0.100–1.458)	0.159	-	-
OTH	76 (6.26)	4 (5.26)	ref	-	-	-
Unknown	16 (1.32)	1 (6.25)	-	-		
Switching regimens						
Yes	259 (21.33)	14 (1.54)	0.516 (0.179–1.484)	0.220	-	-
No	949 (78.17)	28 (2.95)	ref	-	-	-
Unknown	6 (0.49)	0 (0.00)	-	-	-	-
ART adherence						
Good	1,168 (96.21)	28 (2.40)	0.221 (0.063–0.772)	0.018	0.277 (0.070–1.091)	0.067
Poor	30 (2.47)	3 (10.007)	ref		ref	

**Table 5:** Multifactorial analysis of pLLV among HIV-infected patients with low-level viremia in Chongqing, 2019–2023.

Characteristics	All					
	Total	pLLV	OR (95% CI)	*p*	Adjusted OR (95% Cl)	*p*
Age at diagnosis, years						
<20	14 (1.15)	6 (42.86)	1.354 (0.454–4.036)	0.587	2.590 (0.352–19.067)	0.350
20–29	240 (19.77)	47 (19.58)	0.440 (0.289–0.667)	0.000	0.948 (0.431–2.086)	0.894
30–39	298 (24.55)	71 (23.83)	0.565 (0.386–0.825)	0.003	1.008 (0.500–2.033)	0.982
40–49	211 (17.38)	66 (31.28)	0.822 (0.552–1.222)	0.332	1.751 (0.847–3.616)	0.130
50–59	219 (18.04)	68 (31.05)	0.813 (0.548–1.205)	0.302	0.868 (0.449–1.679)	0.675
≥60	230 (18.95)	82 (35.65)	ref		ref	
Marital status						
Married	424 (34.93)	119 (28.07)	1.023 (0.760–1.378)	0.879	0.544 (0.286–1.038)	0.065
Unmarried	225 (18.53)	55 (24.44)	0.849 (0.587–1.227)	0.383	0.830 (0.397–1.732)	0.619
widowed	47 (3.87)	22 (46.81)	2.308 (1.255–4.247)	0.007	0.993 (0.332–2.968)	0.990
Divorced	76 (6.26)	23 (30.26)	1.138 (0.669–1.938)	0.633	0.378 (0.139–1.026)	0.378
Unknown	442 (36.41)	122 (27.60)	ref		ref	
HIV transmission route						
HSX	545 (44.89)	165 (30.28)	0.695 (0.224–2.155)	0.528	0.125 (0.025–0.614)	0.010
MSM	111 (9.14)	29 (26.13)	0.566 (0.171–1.869)	0.350	0.268 (0.051–1.416)	0.121
IDU	13 (1.07)	5 (38.46)	ref	-	-	-
Unknown	545 (44.89)	142 (26.06)	-	-	ref	-
Degree of education						
Primary school or below	163 (13.43)	62 (38.04)	1.714 (1.202–2.444)	0.003	2.052 (1.014–4.194)	0.046
Junior school	189 (15.57)	57 (30.16)	1.206 (0.850–1.710)	0.295	1.830 (0.908–3.689)	0.091
College or above	96 (7.91)	20 (20.83)	0.735 (0.438–1.234)	0.244	1.091 (0.416–2.860)	0.859
Unknown	766 (63.10)	202 (26.37)	ref		ref	
Log_10_ (Baseline VL)						
<3	342 (28.17)	85 (24.85)	0.665 (0.492–0.899)	0.008	0.569 (0.329–0.984)	0.044
3–5	306 (25.213)	68 (22.22)	0.574 (0.417–0.792)	0.001	0.612 (0.351–1.065)	0.082
≥5	566 (46.62)	188 (33.22)	ref		ref	
Treatment regimens						
NRTI + NNRTI	818 (67.38)	210 (25.67)	0.967 (0.567–1.650)	0.902	0.504 (0.201–1.262)	0.143
NRTI + PI	63 (5.19)	23 (36.51)	1.610 (0.7817–3.320)	0.197	0.957 (0.271–3.371)	0.945
NRTI + INSTI	241 (19.85)	78 (32.37)	1.340 (0.752–2.387)	0.321	0.433 (0.165–1.137)	0.089
OTH	76 (6.26)	20 (26.32)	ref		ref	
Unknown	16 (1.32)	10 (62.50)				
Switching regimens						
Yes	259 (21.33)	69 (26.64)	0.923 (0.677–1.258)	0.611	-	-
No	949 (78.17)	268 (28.24)	ref	-	-	-
Unknown	6 (0.49)	4 (66.67)	-	-	-	-
blips/pLLV						
blips	822 (67.71)	28 (3.41)	0.009 (0.006–0.014)	0.000	0.010 (0.005–0.018)	0.000
pLLV	392 (32.29)	313 (79.85)	ref		ref	-

INSTIs, Integrase strand transfer inhibitors; NNRTIs: Non-nucleoside reverse transcriptase inhibitors; NRTIs, Nucleoside reverse transcriptase inhibitors; PIs, Protease inhibitors.

## Discussion

To our knowledge, this represents the first large-scale investigation of clinical correlates in people with HIV-1 and LLV from Chongqing, identifying distinct risk factors for VF and pLLV. Key protective factors against VF included baseline log₁₀VL < 3 and LLV-phase VL < 200 copies/mL, while baseline log_₁₀_VL < 3 also demonstrated protection against pLLV. Lower educational attainment (≤elementary school) emerged as a pLLV risk factor.

The inverse relationship between baseline VL and VF risk aligns with established evidence linking elevated pre-ART viremia to treatment failure (15, 23–26). Contemporary data from INSTI-era cohorts corroborate this pattern, with baseline VL > 1.0E+05 copies/mL associated with the risks of LLV/VF even under integrase inhibitors (27). Our findings extend these observations by demonstrating differential risk stratification across LLV levels: blips (>200 copies/mL) and persistent LLV (50–200 copies/mL) both conferred elevated VF risk (1, 4, 17, 19, 28). These discrepancies likely stem from heterogeneous LLV definitions across studies, particularly regarding the interpretation of blips. In our research, blips were defined as VL measurements within 50–1,000 copies/mL across one-month intervals criterion that differs from conventional within 30 days observation windows used in other cohorts (17). This methodological variation in blips characterization underscores the critical need for standardized virologic monitoring protocols.

Our study identified LLV level as a prognostic determinant, with individuals having a VL > 200 copies/mL demonstrating significantly higher risk of virologic rebound compared to those with VL between 50 and 199 copies/mL. LLV with VL < 200 copies/mL was found to be a protective factor against VF risk. These findings align with those of Hermans LE et al. (12), who reported that LLV (VL of 51–999 copies/mL) significantly increased the risk of subsequent VF (VL > 1,000 copies/mL) in a large multicenter cohort of adults receiving suppressive first-line cART (aHR: 2.6, 95% CI: 2.5–2.8). Tavitiya Sudjaritruk et al. also observed a significantly higher incidence of VF in children with LLV of high VL (400–1,000 copies/mL) compared to those with LLV of low VL (<400 copies/mL) (*p* < 0.001) (29). High-level VL during LLV may serve as an indicator of increased VF risk in these populations. These consistent patterns across populations suggest implementing stricter LLV thresholds (< 200 copies/mL) could optimize clinical monitoring strategies through: (1) Enhanced adherence tracking; (2) Frequent virologic surveillance, and (3) Timely resistance testing.

Subgroup analyses further highlight the heterogeneity of risk factors across distinct populations, revealing nuanced predictors of virologic outcomes. Subgroup analyses highlight the heterogeneity of risk factors across populations. In MSM individuals, the protective role of low LLV and baseline VL aligns with overall trends, but small sample size hinders interpretation of blips and adherence effects, emphasizing the need for larger MSM cohorts. Non-MSM populations show distinct vulnerabilities: moderate baseline VL (3–5 log) emerges as a risk factor, potentially reflecting differences in viral reservoir dynamics or treatment adherence, while sociodemographic factors (e.g., education, marital status) influence pLLV risk, underscoring the importance of tailored support for low-education groups. INSTI-based regimens demonstrate lower VF rates, with adherence showing promise, but blips effects require confirmation in larger samples. Consistently, non-INSTI subgroups validate low baseline VL, low LLV, and iLLV as robust protective markers, reinforcing their universal relevance. These findings support stratified monitoring: prioritizing LLV surveillance in MSM, intensified follow-up for non-MSM with moderate baseline VL, and further investigation of INSTI-specific dynamics to optimize targeted ART strategies.

The protective effect of lower baseline VL (log₁₀ < 3) against pLLV may reflect reduced viral reservoir size, as elevated zenith VL correlates with increased cell-associated HIV DNA (30). This reservoir dynamic necessitates prolonged ART duration for effective suppression, potentially explaining pLLV persistence in patients with high initial viremia (31). The association between limited education and pLLV risk likely stems from multifaceted care continuum challenges: delayed diagnosis, suboptimal ART understanding, and adherence barriers in populations with lower educational attainment. Notably, while suboptimal adherence is an expected mediator, it was not identified as an independent risk factor in our analysis. This discrepancy may arise from study design factors: First, the exclusion of patients with irregular follow-up or missing VL data-a group potentially enriched with adherence challenges and socioeconomic vulnerability—may have diluted measurable adherence effects. Second, adherence assessments relying on self-report and pharmacy records could underestimate true non-adherence, particularly among individuals with lower educational attainment who may not recognize occasional missed doses as significant. Consequently, lower education likely functions as a surrogate marker for socioeconomic barriers (e.g., constrained healthcare access) that contribute to pLLV through mechanisms extending beyond medication-taking behaviors. Based on these findings, we propose two resource-optimized strategies: (1) Implement risk-stratified monitoring for patients with LLV > 200 copies/mL through intensified follow-up and adherence interventions; (2) Strengthen community health worker supervision networks for individuals with primary-level education or less, utilizing on-site medication oversight by community physicians to reduce pLLV risk.

### Study limitations

First, the retrospective design constraints limited data completeness, introducing potential selection bias. Second, Adherence measurement relied on composite self-report and pharmacy records rather than objective methods like electronic monitoring. While this approach is pragmatic for large-scale clinical cohorts, it may overestimate true adherence levels, particularly for marginal adherence cases near the 95% threshold. Third, DNA quantification, a pivotal metric for assessing the magnitude of the viral reservoir, remains absent from routine clinical care in China. As a result, we were precluded from exploring its relationship with LLV and the ensuing clinical outcomes, a significant limitation given the critical role of viral reservoir dynamics in HIV disease progression. Additionally, our study did not assess the influence of co-infections (e.g., viral hepatitis or tuberculosis) on LLV outcomes. Future studies should systematically evaluate comorbidities to determine their impact on LLV persistence and treatment outcomes (32). Finally, while acquired resistance likely mediates LLV-VF progression, future studies need to systematically analyze what types of drug resistance mutations develop when patients have persistent low-level viruses in their blood (33).

## Data Availability

The original contributions presented in the study are included in the article/supplementary material, further inquiries can be directed to the corresponding authors.
